# The Italian project to increase health professionals’ training and awareness on rare diseases

**DOI:** 10.1186/1750-1172-7-S2-A16

**Published:** 2012-11-22

**Authors:** Simona Bellagambi

**Affiliations:** 1 UNIAMO Italian Federation of Rare Diseases, Italy

## Background

The scarce knowledge of rare diseases among family pediatricians and general practitioners who operate at local level and in frequent contact with rare disease patients is seen in Italy as one of the causes leading to delay in diagnosis and to the lack of reference to a centre of competence.

A project aimed at the training of these health professionals was started in February 2009 by the Italian Federation of Rare Diseases UNIAMO with the societies and federations of family paediatricians and general practitioners with the support of FARMINDUSTRIA, Association of Pharmas.

The main aims are to train participants to develop a new diagnostic sensitivity but also in the high complexity care of the RD patient (child or adult), to lay the groundwork for the establishment of a handover protocol which allows the RD patients and their family to benefit from a real continuity of care from pediatric to adult age, being this shift of competence now completely random and to create trainers able to transfer this knowledge and these messages in different contexts, at first regional and then provincial, through the organisation of local educational courses as part of the required upgrading of the different health professionals.

## Method

The format of the courses is developed in two parallel sessions and two plenary sessions. The outset is focused on the concept of network. It is followed by two parallel sessions in which the problem of how to raise awareness on possible diagnosis and all the cross-cutting issues of the daily care are addressed. These issues are dealt with either through lectures of competent specialists in the field of complex disability and through a session of interactive clinical cases where the participants can measure themselves with concrete examples with the guidance of specialist. At the same time the Federation has worked to create and update/enrich a database (http://www.malatirari.it) gathering information on different topics where health professionals are at the same time authors and guarantors of the information on the disease from a scientific point of view but also the users.

## Results

As for January 2012 11 courses took place in 11 different regions with a total of 619 participants. In the current year 6 courses are to be held in other regions.

## Conclusions

The increased knowledge among family doctors and pediatricians will better the quality of life of rare disease patients.

